# ECG Changes in Melanoma Patients Undergoing Cancer Therapy—Data from the ECoR Registry

**DOI:** 10.3390/jcm9072060

**Published:** 2020-06-30

**Authors:** Julia Pohl, Raluca-Ileana Mincu, Simone Maria Mrotzek, Lena Hinrichs, Lars Michel, Elisabeth Livingstone, Lisa Zimmer, Reza Wakili, Dirk Schadendorf, Tienush Rassaf, Matthias Totzeck

**Affiliations:** 1 Department of Cardiology and Vascular Medicine, West German Heart and Vascular Center, Medical Faculty, University Hospital Essen, 45147 Essen, Germany; J.Pohl@uk-essen.de (J.P.); Raluca-Ileana.Mincu@uk-essen.de (R.-I.M.); Simone.Mrotzek@uk-essen.de (S.M.M.); Lena.Hinrichs@uk-essen.de (L.H.); Lars.Michel@uk-essen.de (L.M.); Reza.Wakili@uk-essen.de (R.W.); Tienush.Rassaf@uk-essen.de (T.R.); 2 Department of Dermatology, Medical Faculty, University Hospital Essen, 45147 Essen, Germany; Elisabeth.Livingstone@uk-essen.de (E.L.); Lisa.Zimmer@uk-essen.de (L.Z.); Dirk.Schadendorf@uk-essen.de (D.S.)

**Keywords:** immune checkpoint inhibitor therapy, ECG, QTd, QT dispersion, cardiotoxicity

## Abstract

We aimed to evaluate whether therapy with immune checkpoint inhibitors (ICI) leads to changes in electrocardiogram (ECG) parameters in melanoma patients. We retrospectively examined 41 patients (46% women, age 61 ± 12years) with advanced melanoma (stage III/IV) before and during ICI treatment from our “Essen Cardio-oncology Registry” (ECoR). ECGs were analyzed before and 4–12 weeks after therapy started (follow-up, 90 ± 51 days). Heart rate, PR time, QRS duration and duration of the corrected QT (QTc) interval were recorded. QT dispersion (QTd) was calculated. Heart rate, PR time, QRS and QTc did not differ when comparing values before and after therapy started. QTd was prolonged after therapy started (32 ± 16 ms vs. 47 ± 19 ms, *n* = 41, *p* < 0.0001). Subgroup analyses revealed prolonged QTd in patients that received a combination immunotherapy with ipilimumab and nivolumab (31 ± 14 ms vs. 50 ± 14 ms, *n* = 21, *p* < 0.0001), while QTd in patients with anti–programmed death 1 (PD-1) inhibitor monotherapy did not change after therapy started. QTd is prolonged in patients under ICI combination therapy, potentially signaling an increased susceptibility to ventricular arrhythmias.

## 1. Introduction

About 232,100 cases of cutaneous melanoma are diagnosed every year [1]. Over the past few years, two treatment strategies have revolutionized melanoma therapy. Treatments targeting B-raf proto-oncogene serine/threonine-kinase (BRAF) in combination with mitogen-activated protein kinase MEK 1 inhibitors (MEKI) have improved treatment response and overall survival [1]. Improvement of prognosis in advanced melanoma has been further driven by immune checkpoint inhibitor (ICI) therapies [1]. ICI typically targets cytotoxic T-lymphocyte–associated antigen 4 (CTLA-4) and anti–programmed death 1/ligand-1 (PD-1/PD-L1). The CTLA-4 inhibitor, ipilimumab, and the PD-1 inhibitors, nivolumab and pembrolizumab, or combinations of these agents are applied in patients with advanced melanoma [2,3,4].

ICI therapy typically leads to so called immune-related adverse events (irAE). irAEs often occur within the early phase of therapy (≤12 weeks of therapy), and can affect almost every organ system [5]. Cardiac immune-related complications are rare, but hold the highest lethality rates [5]. The most severe cardiac complication is immune related- (ir-) myocarditis, with lethality rates of 43-46% [6]. Signs of ir-myocarditis include increases in cardiac biomarkers like troponins and NT-proBNP, dyspnea, left ventricular (LV) dysfunction, angina pectoris, signs of congestion and electrocardiogram (ECG) abnormalities [6,7,8]. Malignant arrhythmias as ventricular tachycardia (VT), ventricular fibrillation (VF) and conduction disorders (e.g., bundle branch blocks or complete heart block) are frequent in patients with ir-myocarditis [7,8].

The examination of cancer patients in cardio-oncology departments encompasses the assessment of classical cardiovascular risk factors, imaging techniques like echocardiography, measurement of cardiac biomarkers and ECGs to identify patients at risk for the development as well as the early assessment of cancer-therapy induced cardiotoxicity [9,10]. To date, little is known about parameters that help to identify patients at increased risk for the development of cardiotoxicity due to melanoma therapies [11]. The identification of patients at risk for cancer-therapy induced malignant arrhythmias is of exceptional clinical importance. Prolongation of the corrected QT interval (QTc) is one of the most important indicators of a potential for arrhythmias. Several investigations already demonstrated significant QTc prolongation induced by anti-cancer therapies, especially in anthracycline regimens [12]. However, systematic analyses of QTc prolongation-induced arrhythmias and sudden cardiac deaths (SCD) remain rare [13].

QT dispersion (QTd) is defined as the difference between the maximum and minimum QT interval amongst the different leads of the 12-lead ECG [14]. It is an easily measured electrocardiographic marker which is considered to reflect local differences in the repolarization and recovery time of the myocardium [14,15]. First reports on QTd showed that its prolongation distinguished between long QT syndrome patients with ventricular arrhythmias and those without [14]. Other studies showed that QT dispersion was increased in patients after myocardial infarction and heart failure [16,17]. A recently published meta-analysis showed that QTd has a prognostic role for stratifying myocardial infarction or heart failure patients who are at higher risk of arrhythmic events. Yet, the prognostic role of QTd is not fully established and no prognostic role was found regarding all-cause mortality or SCD in those populations [18].

First reports on anthracycline-induced QTd prolongation were submitted over 20 years ago [19]. Cylcophosphamid therapy also prolonged QTd, and prolonged QTd predicted the occurrence of acute heart failure [20].

Since QTc and QTd prolongation can lead to serious cardiovascular complications, the aim of our study was to investigate whether ICI therapy leads to changes in ECG parameters with special emphasis on QTc and QTd.

## 2. Methods

This retrospective investigation involved 41 consecutive patients with advanced melanoma who were referred to our cardio-oncology unit between January 2018 and December 2019 and were included in our “Essen Cardio-oncology Registry” (ECoR). They presented before and 4–12 weeks after the start of ICI therapy. Patients with preexisting atrial fibrillation and patients with risk factors for QT prolongation such as electrolyte abnormalities (hypokalemia, hypocalcemia or hypomagnesemia) or use of medications associated with QT prolongation were excluded from the study. Anthropometric measurements (height, weight) were done in all patients and body mass index (BMI) was calculated accordingly. Data regarding different risk factors such as hypertension, hypercholesterolemia and premedication were acquired retrospectively from medical records. The ECoR study was approved by the institutional ethics committee of the University of Duisburg-Essen (Essen, Germany—19-8632-BO) and conformed to the principles of the Declaration of Helsinki.

Twelve-lead ECGs were recorded before and after the start of treatment using GE Healthcare ECG machines (GE, Milwaukee, WI, USA). All ECGs were recorded during the morning hours to prevent changes of ECG parameters due to circadian variation. Heart rhythm, PR and QRS duration and the presence of an atrioventricular (AV) block and a left or right bundle branch block (LBBB or RBBB) were assessed. A first-degree AV block was defined as a PR interval of ≥200 milliseconds (ms). A RBBB was defined as a QRS duration of >120 ms in the presence of a typical RBBB morphology; an LBBB was defined as QRS duration of >120 ms in the presence of a typical RBBB morphology. The QT interval was measured by a single experienced reader. Intra-observer variability was calculated with a correlation coefficient of r = 0.95 (*p* < 0.001).

The QT interval was measured from the beginning of the QRS complex to the end of the down slope of the T-wave where it reaches the isoelectric line [21]. QT interval was corrected according to the heart rate using Bazzett’s formula (QTc = heart rate/√RR interval). The difference between the maximum and minimum QT intervals on any of the standard 12-leads on the same ECG was considered as QT interval dispersion (QTd) [22]. As described previously, QTd was not corrected to the heart rate as it was described to be heart rate independent [23].

Continuous variables were shown as mean ± standard deviation (SD). Normal distribution was tested by the Shapiro-Wilk test. For the parameters that showed normal distribution, the paired student *t*-test was used for detection of differences between examined time points. Non-parametric parameters were compared using the Wilcoxon test for dependent variables. A *p*-value of <0.05 was considered statistically significant.

## 3. Results

### 3.1. Baseline Characteristics

Baseline characteristics of the study population are provided in Table 1. The mean age of the patients was 61 ± 12 years. Nearly half of the patients were female (46%). The patients suffered from advanced stage III (29%) or stage IV (71%) melanoma [24]. Nearly half of them received the PD-1 inhibitor, nivolumab, as monotherapy, the other half received a combination therapy consisting of nivolumab and the CTLA-4 inhibitor, ipilimumab. All patients had normal left ventricular ejection fraction (LV-EF) (>50%; mean 60 ± 5%). Most of the patients had normal troponin and NT-proBNP values (Table 1).

### 3.2. No Changes in Heart Rate, PR, QRS and QTc Intervals

**Figure 1:**
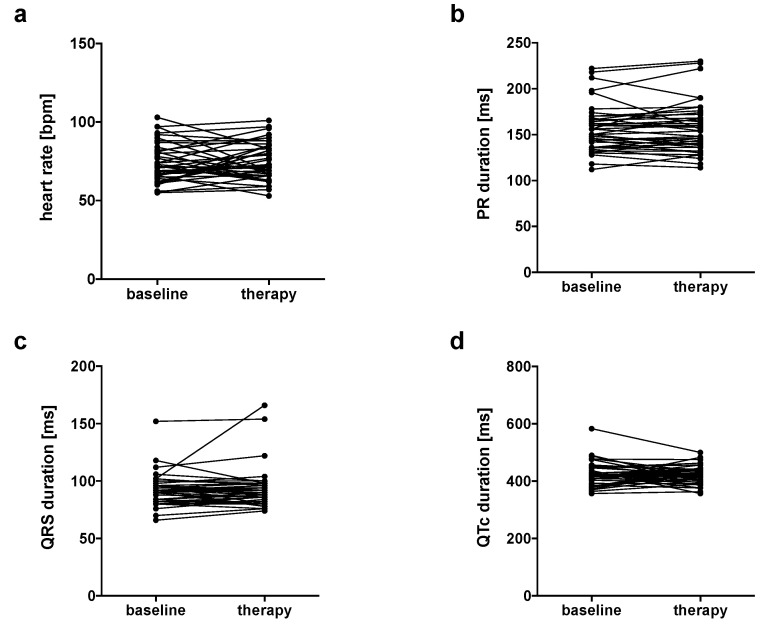
There were no changes in (**a**) heart rate, (**b**) PR duration, (**c**) QRS duration and (**d**) Duration of the corrected QT interval (QTc) comparing values before and during anti-cancer therapy.

Heart rates in the whole study group were normal before therapy started and did not increase or decrease after the start of therapy (74 ± 12 bpm vs. 75 ± 12 bpm, *n* = 41, *p* = 0.6202, Figure 1a). Subgroup analyses revealed that patients who received combination immunotherapy showed increased heart rates after the start of ICI therapy (74 ± 10 bpm vs. 79 ± 12 bpm, *n* = 21, *p* = 0.0367, Table 2). Analyses of the other subgroup did not show changes in heart rates (Table 2). PR intervals did not change after therapy started (whole study group: 157 ± 26 ms vs. 157 ± 28 ms, *n* = 41, *p* = 0.7681, Figure 1b). Only one patient who was treated with PD-1 inhibitor monotherapy developed a new first-degree AV block, none of the patients developed a second- or third-degree AV block (Table 2). QRS intervals were similar when comparing durations before and during therapy (whole study group: 92 ± 14 ms vs. 93 ± 18 ms, *n* = 41, *p* = 0.6431, Figure 1c). None of the patients developed a RBBB or LBBB (Table 2). QTc intervals did not differ when comparing values before and after the start of therapy (whole study group: 428 ± 42 ms vs. 421 ± 31 ms, *n* = 41, *p* = 0.3383, Figure 1d). There were no changes in QTc intervals when comparing patients before and after the start of combination therapy or before and after the start of nivolumab monotherapy (Table 2). All patients had sinus rhythm before and after therapy started, no patient developed new atrial fibrillation or atrial flutter during the follow-up.

### 3.3. Prolongation of QT Dispersion in Patients Undergoing Combination Immunotherapy

**Figure 2:**
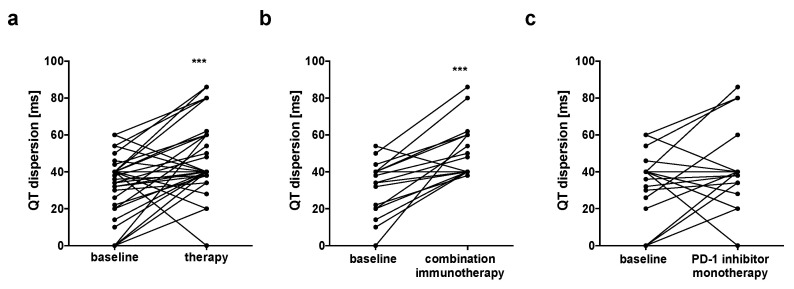
QT dispersion (QTd) increased after therapy start (**a**) QT dispersion in the whole study group increased after start of therapy. Subgroup analyses showed that QTd only increased in (**b**) patients that received combination immunotherapy (*n* = 21), but not in (**c**) patients receiving anti–programmed death 1 (PD-1) inhibitor monotherapy (*n* = 20). *** *p* < 0.001.

QTd was calculated and showed significant prolongation when comparing values before and after therapy started in the whole study group (32 ± 16 ms vs. 47 ± 19 ms, *n* = 41, *p* < 0.0001, Figure 2a/Table 2). Importantly, patients under betablocker therapy showed QTd prolongation after therapy started likewise (26 ± 17 ms vs. 45 ± 19 ms, *n* = 12, *p* = 0.0126). Looking at the subgroups, only patients who were treated with ICI combination therapy showed significant increase in QTd after therapy started (31 ± 14 ms vs. 50 ± 14 ms, *n* =21, *p* <0.0001, Figure 2b/Table 2), while QTd was similar when comparing patients before and after the start of PD-1 inhibitor monotherapy (32 ± 18 ms vs. 40 ± 21 ms, *n* =20, *p* = 0.1099, Figure 2c/Table 2).

## 4. Discussion

We provided findings on the ECG data of 41 patients, from our ECoR database, with melanoma undergoing ICI therapy. Heart rate, QRS, PR and QTc intervals did not change before and after the start of therapy. QT dispersion was prolonged in patients who received ICI combination therapy consisting of nivolumab and ipilimumab, while other treatments did not influence QT interval dispersion.

Stability of heart rate, QRS and PR intervals were not surprising since most of the anti-cancer therapies did not show to affect these parameters. Resting heart rate was shown to be elevated in treatment-naïve cancer patients, and higher heart rates before anti-cancer therapy started are associated with higher mortality rates [25,26]. Similar data on heart rates after or during anti-cancer therapies are not published yet. Development of a LBBB or RBBB can occur during anti-cancer therapies, but reports remain rare [27]. Complete heart blocks have been described in single cases of doxorubicin-induced cardiotoxicity [28] and seem to be common in ir-myocarditis [29,30].

Prolongation of the QT interval is one of the common manifestations of acute and subacute chemotherapy-induced cardiotoxicity, but it is not typical for ICI therapies [31].

QTd is an easy, inexpensive and practical parameter that is thought to be associated with repolarization heterogeneity and the arrhythmogenic potential of several medications. Identification of the exact role of QTd in arrhythmogenesis remains challenging, but many studies have shown that prolonged QTd is associated with the genesis of malignant arrhythmias in patients with myocardial infarction or heart failure [18]. Prolonged QT dispersion was even shown to predict acute heart failure in patients after high-dose cyclophosphamide therapy [20]. Although previous studies have shown high inter- and intraobserver variability in measurements of QTd, with relative errors of approximately 25–40% variability in manual measurements of QTd [23], the authors concluded that QT dispersion was a sensitive enough index to show abnormalities even earlier than on echocardiography [20]. Besides variability in measurements, QTd measurements were critically discussed concerning its circadian variation [32,33] and concerning the assessment from various ECG leads (e.g., all 12 leads, only precordial leads, only three leads, only limb leads). However, the definite regimen for QTd assessment remains unclear, but one could assume that using more projections of the T loop into different leads results in more sensitive measurements [23]. Still, the most important part seems to be using identical methods in all patients belonging to a study cohort. The exact mechanisms of ICI combination therapy induced prolongation of QT dispersion are unclear. Our data are in line with a previous study investigating ECG changes after nivolumab treatment in solid tumors [31] and confirm that the combination of CTLA-4 and PD-1 inhibitors increases the risk for the development of cardiac irAEs when compared to monotherapy [30]. Our data point in the same direction and seem to underline the possible cardiotoxicity of combination immunotherapies contributing to a potential ventricular repolarization instability. ECG evaluation is of special interest in ICI therapy patients considering the fact that most of the patients with ir-myocarditis show changes in ECG parameters and biomarkers even before changes in left ventricular ejection fraction [6].

The study has several limitations. First, the data was analyzed retrospectively. Second, this study only comprises data of 41 patients. Third, with respect to our observed findings in QTd variation, it has to be noted that we did use standardized measurements of all 12 ECG leads and did not specifically investigate the role of different vectors or specific lead combinations. Nevertheless, since all measurements were carried out in the same way, we believe that these findings are robust and hypothesis-generating, serving as a basis for future prospective studies investigating the role of repolarization changes in the patients in more detail. The present study did not aim at investigating the effects of prolonged QTd on the occurrence of cardiotoxicity such as heart failure or malignant arrhythmias. It was developed to assess whether ICI therapies influence ECG parameters. QTd prolongation probably predicts the occurrence of cardiovascular events, but further studies will be required to confirm the impact of ICI combination or other therapies on QTd, and prospective long-term follow-up data will be needed to verify its impact on occurrence of clinical events in ICI therapies.

In view of our data, accurate cardiologic assessments of melanoma patients undergoing ICI combination therapies should be advised on a regular basis, and patients with increased QTd might undergo further continuous ECG monitoring and reevaluation, e.g., by Holter monitoring.

## Figures and Tables

**Table 1:** Baseline characteristics.

Parameters	All (*n* = 41)	Combination Immunotherapy (*n* = 21)	PD-1 Inhibitor Monotherapy (*n* = 20)
Age (years), mean ± SD	61 ± 12	61 ± 14	60 ± 11
Female sex (%)	46	48	45
BMI (kg/m^2^), mean ± SD	27.3 ± 4.2	26 ± 3	28.7 ± 4.9
Hemoglobin (g/dL), median (range)	13.1 (8–16)	13.7 (10–16)	13.7 (10–16)
Platelets (×1000/µL), median (range)	274 (154–577)	263 (234–577)	273 (161–549)
Creatinine (mg/dL), median (range)	0.9 (0.7–1.4)	1 (0.7–1.4)	0.9 (0.5–1.1)
CRP (mg/dL), median (range)	0 (0–18)	0 (1–18)	0 (0–4)
Troponin (ng/L), median (range)	5 (0–114)	0 (0–114)	0 (0–17)
NTproBNP (pg/mL), median (range)	70 (9–848)	87 (9–512)	51 (20–848)
Comorbidities			
Arterial hypertension (%)	41	38	45
Diabetes (%)	5	5	5
Smoking (%)	24	14	10
Previous stroke (%)	7	10	5
Known CAD (%)	7	0	15
Known CHF (%)	10	10	10
Premedication			
ACE-I/ARB (%)	32	29	35
Betablocker (%)	29	29	30
Statins (%)	7	5	10
Aspirin/DAPT (%)	22	5	20
Tumor stadium III (%)	29	38	20
Tumor stadium IV (%)	71	62	80
Therapy regimen			
Nivolumab monotherapy (%)	46	0	100
Nivolumab/Ipilimumab (%)	53	100	0

BMI: body mass index; CRP: C-reactive protein; CAD: coronary artery disease; CHF: congestive heart failure; ACE-I: angiotensin-converting enzyme inhibitor; ARB: angiotensin receptor blocker; DAPT: dual antiplatelet therapy.

**Table 2:** Electrocardiogram (ECG) data.

ECG Parameters	All (*n* = 41)			Combination Immunotherapy (*n* = 21)			PD-1 Inhibitor Monotherapy (*n* = 20)		
	Baseline	Therapy	*p*	Baseline	Therapy	*p*	Baseline	Therapy	*p*
Heart rate (bpm)	74 ± 12	75 ± 12	0.6201	74 ± 10	79 ± 12	0.0367	73 ± 15	70 ± 10	0.3283
PR interval (ms)	157 ± 26	157 ± 28	0.7681	157 ± 25	155 ± 25	0.4475	156 ± 27	160 ± 31	0.0495
1st degree AV block (%)	7.3	7.3	1.0000	4.8	4.8	1.0000	5	10	1.0000
2nd degree AV block (%)	0	0	-	0	0	-	0	0	-
3rd degree AV block (%)	0	0	-	0	0	-	0	0	-
QRS interval (ms)	92 ± 14	93 ± 18	0.6431	91 ± 13	93 ± 20	0.6736	94 ± 15	94 ± 16	0.8557
LBBB (%)	0	0	-	0	0	-	0	0	-
RBBB (%)	0	0	-	0	0	-	0	0	-
QTc interval (ms)	428 ± 42	421 ± 31	0.3383	432 ± 50	424 ± 32	0.4055	423 ± 32	419 ± 30	0.6240
QTc > 450 ms (%)	24.3	14.6	0.7284	33.3	14.3	0.7174	20	15	1.00
QTd (ms)	32 ± 16	47 ± 19	<0.0001	31 ± 14	51 ± 14	<0.0001	32 ± 18	41 ± 21	0.1099

bpm: Beats per minute; LBBB: Left bundle branch block; RBBB: Right bundle branch block. QTc: corrected QT interval.
